# Perspectives on the upgradation of Gleason score after radical prostatectomy: Why our uropathologists need to remain abreast with current concepts

**DOI:** 10.4103/0970-1591.70599

**Published:** 2010

**Authors:** Gagan Gautam

**Affiliations:** Department of Surgery, University of Chicago Medical Center, Chicago, IL, USA

Dear Editor,

I read with interest the manuscript by Nayyar *et al*. on the upgradation of Gleason score in radical prostatectomy specimens in relation to the tissue obtained from preoperative needle core biopsies (NCB).[1] According to this well-designed study, 177 patients undergoing radical prostatectomy from 2002 to 2008 had their final Gleason score upgraded from 5.51 ± 1.52 (range 2–9) to 6.2 ± 1.42 (range 2–9) after radical prostatectomy. The authors correctly concluded that this unreliability of preoperative assessment casts a doubt on our ability to offer surveillance protocols to patients with low Gleason scores on preoperative biopsy and may render clinical comparisons between various treatment modalities difficult.

In my view, however, this study also highlights the need to have dedicated uropathologists coordinating with urology teams to administer the best possible diagnostic care to the patients. It has been well established since a while now that a Gleason score of 2–4 should never be diagnosed on NCB with recent suggestions that even a Gleason score of 5 is becoming out of bounds for pathologists interpreting these specimens. These suggestions, which were first espoused by Epstein in 2000, became established in the 2005 consensus conference of the International Society of Urological Pathology (ISUP). The reasons for these conclusions include the following: poor reproducibility even among experts, upgradation of almost all cases after prostatectomy, inability to assess the edge of the lesion, and propagation of a misconception that the urologist may be dealing with an “indolent” tumor. In the year 2001, only 2.4% of all pathologists surveyed, were diagnosing Gleason score 2–4 on NCB. Accordingly, the proportion of biopsies reported as Gleason 2–4 also decreased from 2.7% to 0% after this conference.[2]

Well, apparently not.

This study is another case in point that we as urologists and urooncologists need the support of our pathology colleagues, who in turn owe it to our patients to stay abreast with the latest in uropathologic literature and update their diagnostic services in line with the ever-changing standard of care.
